# Male Takeovers Are Reproductively Costly to Females in Hamadryas Baboons: A Test of the Sexual Coercion Hypothesis

**DOI:** 10.1371/journal.pone.0090996

**Published:** 2014-03-12

**Authors:** Pablo Polo, Victoria Hernández-Lloreda, Fernando Colmenares

**Affiliations:** 1 Grupo UCM de Estudio del Comportamiento Animal y Humano, Madrid, Spain; 2 Departamento de Psicobiología, Facultad de Psicología, Universidad Complutense de Madrid, Campus de Somosaguas, Madrid, Spain; 3 Departamento de Metodología de las Ciencias del Comportamiento, Facultad de Psicología, Universidad Complutense de Madrid, Campus de Somosaguas, Madrid, Spain; University of Arizona, United States of America

## Abstract

During male takeovers, in addition to fighting off the female’s current mating partner, males may exhibit intense aggressive mate guarding of the newly acquired females. Recent studies indicate that coercive sexual aggression by males is an important strategy through which sexual conflict is expressed. Previous tests of the sexual coercion hypothesis in primates have focused on assessing if female mate choice is effectively reduced by male aggression, however, only one recent study has tested a critical prediction of this hypothesis, namely, that male coercion is reproductively costly to victim females. The present study uses 15 years of data on inter-birth intervals from a large multilevel colony of baboons, mostly *Papio h. hamadryas*, with a mating system based on harem-defence polygyny to examine if male takeovers impact the length of the abducted females’ inter-birth intervals. Our analysis of 121 inter-birth intervals from 45 adult females indicates that male takeovers are reproductively costly to abducted females as they are associated with an increase in the time they take to conceive and a lengthening of the inter-birth intervals. We discuss how several factors may contribute to this reproductive cost, including male-female sexual conflict, male-male competition, and female-female competition. Our findings suggest that the male’s aggressive herding is the main contributor to the abducted females’ immediate reproductive cost. We argue that although some of the male’s aggressive herding may be driven by male-male competition, nonetheless, it serves a coercive function as it both constrains the female’s mate choice options and hampers her immediate breeding performance. This conclusion is backed up by results obtained in the only other study that has tested the same prediction and which has been carried out in a wild population of hamadryas baboons.

## Introduction

Darwin [1] conceived sexual selection as consisting of two major mechanisms: intra-sexual competition in a scenario of limited breeding opportunities, and intersexual selection or choice among unequally attractive and valuable mating partners [2]. Mainstream views acknowledge that many sexually dimorphic traits displayed by both sexes may also reflect the operation of a third evolutionary force, namely, intersexual conflict (or conflict between the sexes) [3]–[6]. Sexual conflict theory claims that mating partners have both overlapping as well as divergent reproductive interests, as males and females generally differ in their reproductive rates and, therefore, in the way they can maximize their reproductive success by overcoming sex-specific constraints [7], [8]. As a consequence, some sexually dimorphic traits have probably evolved in response to conflicts between the sexes, especially if the partners’ divergent optima cannot be achieved simultaneously [3], [6]. In this scenario, sexually antagonistic selection will emerge and may drive an arms race of strategies and counterstrategies between the sexes that can be expressed at different levels of biological organization (e.g. genomic, morphological, physiological, and behavioural) and at different stages throughout the reproductive process (e.g. prior to or after mating, during pregnancy, during lactation). At a behavioural level, conflicts between the sexes can be expressed via sexual coercion by males. Smuts and Smuts ([9] pp. 2–3) defined sexual coercion as the “use of force, or threat of force, that functions to increase the chances that a female will mate with him at a time when she is likely to be fertile, and to decrease the chances that she will mate with other males, at some cost to the female”.

There is growing recognition that sexual conflict is a significant evolutionary force [3]–[6], [10]. However, the majority of empirical studies of sexual conflict have been done on invertebrates (for a review see [3]). Recently, researchers from a number of disciplines including primatology, evolutionary anthropology, and behavioural biology, have started to investigate sexual conflict and sexual coercion in humans and in nonhuman primates [11]–[13]. Among mammals, anthropoid primates are salient in that they display a suite of characteristics that make them especially prone to evolve sexually dimorphic traits via sexual conflict and sexual coercion. These include their multi-male, multi-female social systems; their promiscuous or polygynous (harem-defence) mating systems; their remarkable sexual dimorphism in body size, weaponry and other elaborate ornaments; their long lactation periods during which they lack ovulatory cycling; their exposure to immigrant, potentially infanticidal males; and their asymmetries in levels of parental investment and reproductive rates [13]–[15].

The sexual coercion hypothesis posits that males direct aggression toward females as a sexually selected strategy to control female sexuality. More specifically, male aggression against females (and their young) qualifies as sexual coercion if it (1) increases the aggressor male’s mating and reproductive success, (2) suppresses the victim female’s opportunity to choose her mating partners via limiting her promiscuity or via forcing her to mate with non-preferred males, and (3) imposes fitness costs on the victim female (e.g. [9], [16]). Indeed, conditions two and three reflect intersexual conflict as the male’s behaviour is harmful to the victim female’s reproductive interests.

Recent studies of sexual coercion by primate males have concentrated on just a few species, the chimpanzee [17], [18], the gorilla [19], the chacma baboon [20], and the hamadryas baboon [21], [22]. However, these differ from one another along several dimensions, including the bonding system, the mating system, the dispersal and transfer patterns, and the mode of transfer [23]–[25]. Thus, the hamadryas baboon *(Papio h. hamadryas)* is cross-bonded; males monopolize a semipermanent harem of females (i.e., female defence polygyny) that they guard aggressively (via herding behavior) at all times, not only when they are in the fertile period of their menstrual cycle, and females are forcibly and aggressively transferred across reproductive units during male takeovers [26]–[29]. These and other studies show that aggressor males do benefit from practicing sexual coercion, and that the victim females’ reproductive decisions are significantly constrained as a consequence. One of the key criteria for a male’s aggressive behaviour to qualify as sexual coercion is that it imposes fitness costs on the females [9], [16]. These may come in two currencies, survival costs and reproductive costs. Some studies indicate that victim females can sometimes be injured or even killed by males in this context [16]; in other words, that male coercion may jeopardize the victim female’s survival. Also, infanticide, as the extreme expression of male coercion, does impose a tremendously severe cost on a female’s breeding output [30]. However, so far there has been only one recent study that has investigated if male coercive guarding may impose reproductive costs on females other than those caused by infanticide [22]. This study has been carried out by Swedell et al. [22] in a wild population of hamadryas baboons *(Papio h. hamadryas)* and they reported that after incurring takeovers, abducted females experienced a significant increase of the interval between surviving infants.

Male takeovers, broadly defined as events in which one male appropriates one or more already mated, breeding females, are known to be one of the most common reproductive tactics whereby young males start their reproductive careers and fully-grown adult males further increase mate number and mating success [14], [31]. Although male takeovers are a widespread reproductive strategy to gain sexual access to fertile females (e.g. [14], [31]), in the particular case of the hamadryas baboon [21], [22], [28], [32]–[34], and other harem-forming species (e.g. gelada baboon [35], gorilla [36]), male takeovers are events in which researchers can potentially witness the whole set of processes that drive sexual selection, that is, male competition, female choice, sexual conflict (and sexual coercion) and female competition. Moreover, in the hamadryas baboon these events are associated with a significant increase of the male’s herding behaviour towards the newly acquired female [21], [33], which has led some workers to see these female transfers between breeding units as a coercive male reproductive strategy (i.e. sequestration) rather than as a female reproductive strategy [16], [21].

The goal of the present study is to determine whether male takeovers in the hamadryas baboon impose reproductive costs on the newly acquired females. These reproductive costs were assessed by measuring the length of the inter-birth interval (or IBI), a key life-history variable [15], in females that resumed sexual cycling after the postpartum amenorrhea period under three conditions: when they stayed on in the same one-male unit (or OMU) without any demographic changes, when they remained in an OMU that received one (or more) new female(s) during a male takeover, and when they were taken over by a new male and forcibly transferred into the latter’s OMU. Under the sexual coercion hypothesis, the length of the IBI was expected to be longer when females were transferred than when they remained with the same male. More specifically, we predicted that newly acquired females would take a greater number of cycles to conceive in response to a male takeover than if they would mate with their former male and, therefore, would have lengthened IBIs. Thus, this study provides a strong test of the sexual conflict theory in a vertebrate species by examining if the male’s coercive behaviour during male takeovers negatively impacts the abducted females’ breeding performance.

## Materials and Methods

### Ethics Statement

The present study was non-invasive and strictly adhered to the legal requirements of Spain. No approval from any Research Committee was necessary because no special permission for the use of animals in purely observational studies is required in Spain. Animal husbandry and research in the Madrid Zoo comply with the “EAZA Minimum Standards for the Accommodation and Care of Animals in Zoos and Aquaria” and the “WAZA Ethical Guidelines for the Conduct of Research on Animals by Zoos and Aquariums”. The Madrid Zoo and Aquarium granted permission to carry out this study.

### Baboon Colony and Study Sample

The study was carried out in a large colony of baboons housed at the Madrid Zoo. The colony was established in 1972 and was studied uninterruptedly until 2001. Throughout the 30-year period, long-term data were collected three or four days weekly on the females’ reproductive life history, the membership of one-male/multi-female reproductive units (OMUs), the observed copulations, and the males’ social and reproductive trajectories. The latter included records of the males’ social status (i.e., young follower, young leader, prime leader, old leader, and old follower) and the number of reproductive females they mated with throughout their lifespan (e.g. [37], [38]). All colony individuals (up to 340 over the years) were recognized individually. The Madrid colony of baboons can be considered as consisting of two bands: band I, from June 1972 to April 1985, and band II, from April 1985 to August 2001, based on the major manipulation that occurred in 1985, when all resident adult males were removed and three novel adult males were introduced [39]. Both bands reproduced the multilevel social system that has been described for hamadryas baboons, *Papio h. hamadryas,* in the natural habitat (Madrid, band I [37], Madrid, band II [40]; Ethiopia, Erer-Gota [41], Ethiopia, Filoha [42]). Band I consisted mostly of hamadryas baboons, a few yellow baboons *(P. h. cynocephalus)*, and a few hybrids between these two subspecies, whereas band II comprised hamadryas and some hamadryas-anubis *(P. h. anubis)* hybrids [39]. It is important to note that the colony deployed the harem-defence polygynous mating system so characteristic of the hamadryas baboon (e.g. [28], [32], [41]). The baboon colony was housed in a large enclosure consisting of an outdoor compound with an attached indoor area (e.g. [39]). The outdoor compound was a large pit (36 m long, 26 m wide, 7 m deep) which was made up of flat surfaces at multiple levels, and several climbing structures and boards. Inside the compound there was also a water-filled moat from which 21 flat-circular islands emerged. The indoor area consisted of four rooms which were inaccessible to zoo visitors. This area could be freely accessed by the animals at any time, day and night. Water was available ad libitum and food was provided twice a day. The present study uses data from band II only; they were collected by FC with the assistance of several students under FC’s guidance and supervision.

### Measuring Reproductive Success

A way to estimate a female’s reproductive success is to measure the average time it takes for her to produce two consecutive offspring. This period is referred to as the length of the inter-birth interval (IBI), which is made of three components: the length of gestation, the length of the amenorrhea postpartum period and the time to conception. In this study we use the time to conception and the length of the females’ IBIs as measures of their breeding performance. In this species, follicular activity is associated with changes in the swelling and brightness of the red/pink colour of the skin covering the perineum, genitalia and adjacent areas (for a review, see [43]). The luteal phase of the female’s reproductive cycle begins rather abruptly with the onset of detumescence of the sexual skin, a loss of colour, and an increase of wrinkles, all of which are highly noticeable signals. Females lack sexual swellings throughout pregnancy and the postpartum lactation period. We use these criteria to estimate conception date (i.e. 2 days before onset of detumescence [44]), resumption of ovarian activity following the postpartum amenorrhea period, and follicular activity generally. Given our observation protocol (see above) the error margin for recorded birthdates was up to +3 days.

### Data Analysis

The length of a female’s IBI can be influenced by a number of factors. Thus, for each IBI record, we computed the female’s age, the unit’s size (i.e. number of females in the OMU), whether or not the offspring survived to weaning age (estimated to be 200 days on average, unpublished data), and whether the female changed OMUs due to a male takeover. Females were classified into one of three possible demography-related status categories. *Abducted females* were those who changed OMUs; *involved females* were those who belonged to OMUs that were enlarged by the entry of one or several abducted females via male takeovers; and, finally, *uninvolved females* were those who belonged to OMUs that did not experience a takeover and, therefore, whose demographic status remained unchanged throughout the female’s IBI. This categorization allowed modelling the impact of a male takeover on the abducted females’ IBI while controlling for the effects of other factors that may operate as a consequence of the takeover and may affect all of the females in the unit that receives the newly acquired female (e.g. female competition or male-to-female redirected aggression). IBIs of females attached to OMUs that lost one or more females due to male takeovers were not considered in this study because adding a fourth female category had the undesirable effect of increasing the complexity of the model and the category was irrelevant to the aims of this study anyway. Of the 156 IBIs recorded between 1985 and 2001 (i.e., 15 years and 8 months), 35 did not fit into the categories defined above. The final sample available for the analysis consisted of 121 IBIs from 45 adult females, representing 208.1 female-years. The thirty-nine male takeovers analysed in this study took place when the abducted female was already cycling.

Due to the hierarchical structure of the data – a variable number of repeated IBI records per female – we applied two-level hierarchical linear models [45], [46]. This method allowed us to include all the observations from each female while controlling for the lack of independence of observations within individuals. Length of IBIs and time to conception were level-1 units, and females were level-2 units. Female status (or FS, i.e. abducted, involved or uninvolved) and infant survival (or IS, i.e. whether it did or did not survive to weaning age) were entered as level-1 factors and female age (or FA) and unit size (or US) were entered as level-1 covariates. We did not enter parity in the analysis as this variable was strongly correlated with age (r = 0.943, N = 112, P< 0.001). We followed a step-up strategy in order to choose the simplest model that provides the best fit to the observed data [47]. The two-way interactions between the predictor variables were considered when selecting the best fitted model. We compared the fit of nested models with likelihood-ratio tests and used the Akaike information criterion (AIC) to compare non-nested models [47]. We specified full maximum likelihood estimation (ML) and Type III variance. Post-hoc tests (using Bonferroni correction) followed whenever a significant effect was detected. The Pseudo R^2^ index was used to estimate the percentage of variance explained by the final model [48]. We checked for normality of the residuals and for homogeneity of variance in level-1. These assumptions were met when the data were logarithmically transformed. The global significance level was set at α = 0.05. Models were adjusted with HLM v6.08 and post hoc comparisons were performed with IBM SPSS 19 software.

## Results

### Inter-birth Interval

**Figure 1:**
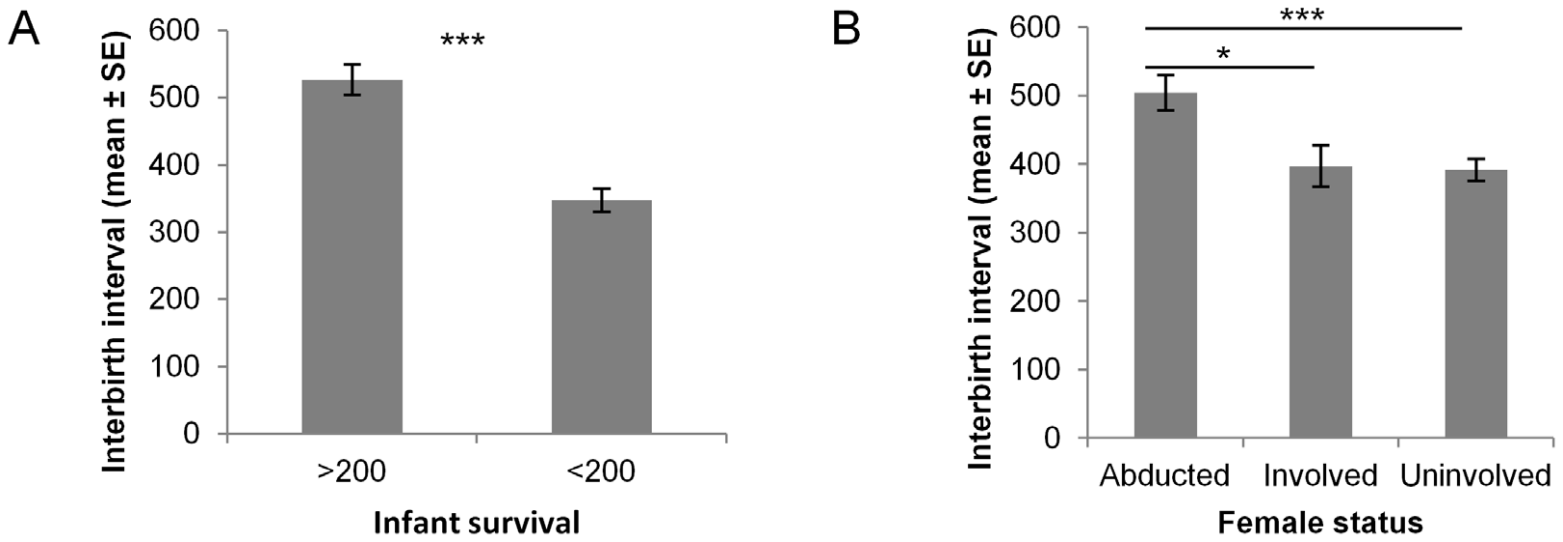
Effects of infant survival and female demographic status on inter-birth intervals. Bars represent the mean±standard error (SE) of the length of the inter-birth interval (A) of females as a function of whether or not infants survived to weaning age (i.e. > 200 vs < 200 days) and (B) for abducted, involved and uninvolved females, respectively. Values of the dependent variable are represented in days although means and standard errors were calculated from the transformed variable (ln[days]). Females showed longer IBIs when infants survived to weaning age. In addition, the IBIs of the abducted females were longer than those of involved and uninvolved females. Asterisks represent significant values. *P<0.05, ***P<0.001.

Table 1 shows the final model fitted to the data on the length of IBIs. The fitted model accounts for 46.6% of the intra-IBI level-1 variance. According to the model’s estimations, the IBIs were 5.96 months longer on average when females succeeded in weaning their infants (mean±SE difference = 0.416±0.055, df = 119.181, p<0.001; Fig. 1A). And, more relevant, regardless of the infant survival, the IBIs of abducted females were 3.57 months longer than were the IBIs of involved females (0.239±0.088, df = 116.2, p = 0.023; Fig. 1B) and 3.75 months longer than were IBIs of uninvolved females (0.253±0.059 df = 108.3, p<0.001; Fig. 1B). In contrast, the length of IBIs of involved females did not differ from those of uninvolved ones (0.014±0.083, df = 112.5, NS).

**Table 1:** Inter-birth interval model.

*Fixed effect*	Estimate	*t*-ratio		*p*
Intercept	6.430	115.624		<0.001
IS = 0	−0.416	−7.497		<0.001
FS = 0	−0.253	−4.267		<0.001
FS = 1	−0.239	−2.715		0.008
***Covariance parameter***	**Estimate**	**df**	*χ* ^2^	***p***
Intercept	0.015	44	69.606	0.008
Residual	0.078			

Estimates of fixed effects and covariance parameters. Predictor variables: IS (infant survival): [0 = the infant did not survive to weaning age, reference category = the infant did survive to weaning age] and FS: [0 = uninvolved females, 1 = involved females, reference category = abducted females].

When IS and FS were adjusted independently (reduced models) we found that IS explained 39.5% of the intra-IBI level-1 variance, whereas FS accounted for 12.3% of that variance.

### Time to Conception

**Figure 2:**
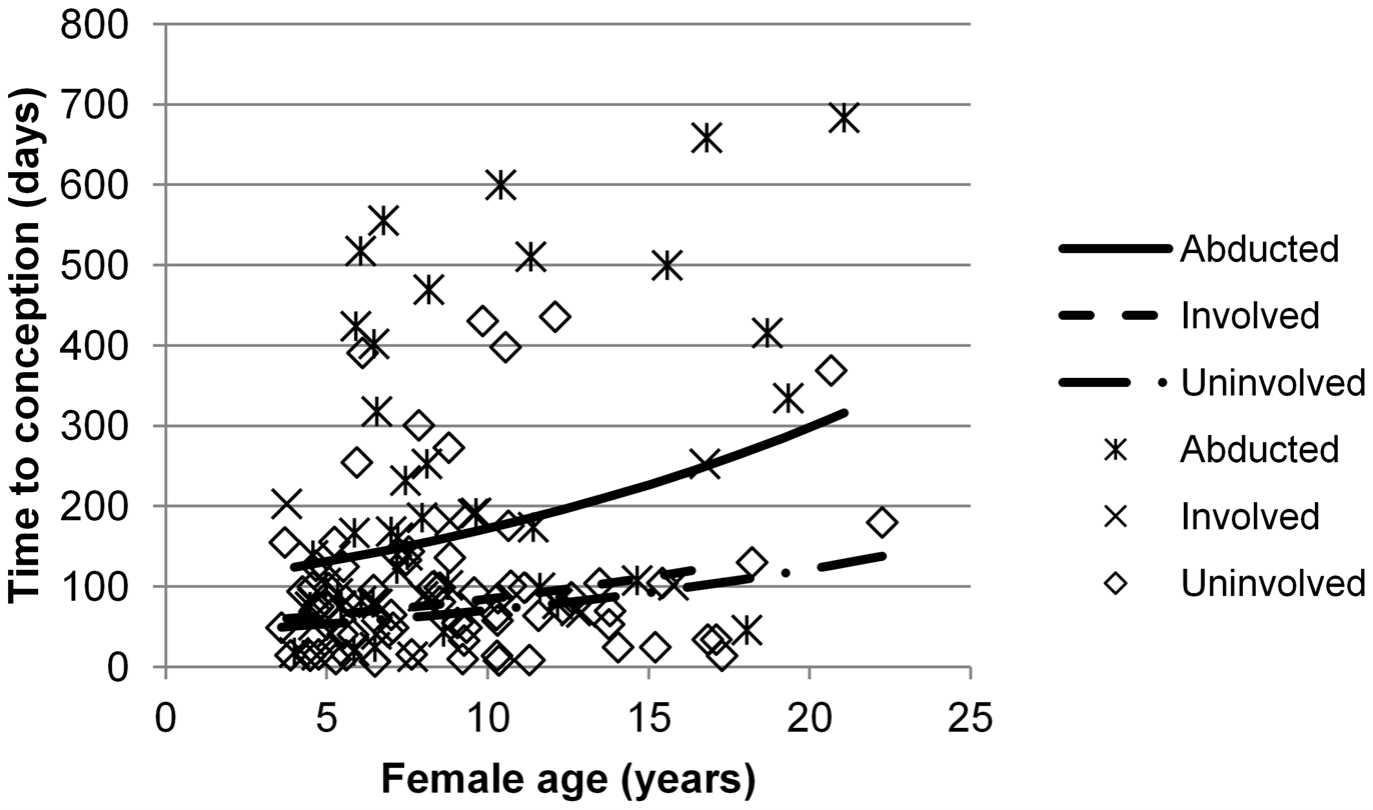
Effects of female age and demographic status on time to conception. Each data point depicts an observed value of time to conception for an individual female according to her demographic status. Abducted, involved and uninvolved females are depicted by asterisks, crosses and squares, respectively. Lines represent expected values along the observed range for each category of female status. Abducted, involved and uninvolved females are represented by a full line, a discontinuous line and a discontinuous and dotted line, respectively. Values of the dependent variable were represented in days, although linear adjustment was calculated from the log-transformed variable. There were differences between the slopes of abducted and uninvolved females and between the slopes of abducted and involved females.

Table 2 shows the final model fitted to the data on time to conception. The fitted model explained 23.9% of the intra-individual variance of time to conception level-1 variance. According to the model’s estimations, abducted females took an extra 2.81 months to conceive compared to involved females (mean±SE difference = 0.710±0.284, df = 118.4, p = 0.041, Fig. 2) and 3.27 months longer than uninvolved females (0.896±0.192, df = 113.9, p<0.001), when these differences were evaluated at the mean female age. Differences between involved and uninvolved females were not statistically significant (0.186±0.266, df = 115.9, NS). Also, time to conception was longer with increasing FA (γ = 1.5×10^−4^, df = 84.0, p = 0.011).

**Table 2:** Time to conception model.

*Fixed effect*	Estimate	*t*-ratio		*p*
Intercept	4.602	18.460		<0.001
FS = 0	−0.896	−4.673		<0.001
FS = 1	−0.711	−2.503		0.014
Female age	1.5×10^−4^	2.602		0.011
***Covariance parameter***	**Estimate**	**df**	*χ* ^2^	***p***
Intercept	0.087	44	54.778	0.128
Residual	0.849			

Estimates of fixed effects and covariance parameters. Predictor variables: IS (infant survival): [0 = the infant did not survive to weaning age, 1 = the infant did survive to weaning age] and female age.

When FS and FA were adjusted independently (reduced models) we found that FS explained 14.1% of the intra-IBI level-1 variance, whereas FA accounted for 7.4% of that variance.

## Discussion

Male herding is a conspicuous signature of the hamadryas male’s behaviour directed at females [27], described as early as 1932 [49]. It is known that in the hamadryas baboon male herding is more frequent when the target female is in the follicular phase of her menstrual cycle [21], [50]. It is also known that herding increases a male’s chances of successfully monopolizing sexual access to females (e.g. [21], [33]) and therefore, by preventing them from mating promiscuously, it curtails their options to play a confusion paternity strategy. More recently, herding behaviour has been identified as a possible coercive strategy used by males during takeovers to condition females to remain spatially close to them and away from their former leader [21], [33]. Lastly, it has also been established that females may be physically harmed as a consequence of this form of coercive mate guarding by males [27], [32]. However, the strong test of whether male takeovers and herding behaviour function as aggressive coercion because they impose reproductive costs to abducted females has been tackled only recently [22].

In this paper, we have also tested this prediction by analysing the effect of male takeovers and associated aggressive herding by hamadryas males on the victim female’s length of the inter-birth interval. The results of our analyses confirm previous findings concerning the impact of raising infants to weaning age on the mother’s length of the IBI [22], [51], [52], and lend further support to the view that male infanticide is clearly reproductively advantageous to males as it eliminates the barrier that delays the mothers’ resumption of ovarian activity [30]. More importantly, our findings reveal that male takeovers are reproductively costly to females, because females abducted during takeovers take longer to conceive and therefore have significantly lengthened IBIs. Zinner and Deschner [34] and Swedell et al. [22] also found that after male takeovers, hamadryas females needed more cycles to conceive, however, whereas in Zinner and Deschner’s study the abducted females’ did not lengthen their IBIs, in Swedell et al.’s study (like in the present study) they did significantly increase their IBIs. (The lack of a lengthening of the abducted females’ IBIs in Zinner and Deschner’s study may well have been due to the small sample size available for analysis, i.e., 9 females altogether.) These results raise at least two critical issues of broad theoretical interest. Firstly, what goes on around male takeovers that make females conceive later and therefore lengthen their inter-birth intervals? Secondly, and related to the latter, can male herding during takeovers be seen as sexual coercion?

Male takeovers are generally stressful and potentially violent events because male challengers have to fight off the female’s current mating partner, have to control the newly acquired female’ movements via aggressive mate guarding, which impacts time budgets, and may attack and kill the females’ dependent offspring [22], [28]. In addition, male takeovers and the transfer of new females into an established OMU may increase female scramble or contest competition within the OMU for access to food resources and to the limited but valuable fitness-enhancing services provided by the unit male [33], [40]. Therefore, male-male competition, intersexual conflict, and female-female competition are all sources potentially contributing to the observed reproductive cost incurred by abducted females during male takeovers. Our results indicate that unit size, a proxy used to assess female competition, is neither a significant predictor of the length of the IBI, nor of the time the abducted females take to conceive again. As a matter of fact, our results show that only the abducted females’ reproductive performance is negatively affected by male takeovers; resident females’ breeding performance is unaffected. It thus seems that the reproductive cost experienced by abducted females is most likely associated with the male’s herding behaviour, which is exacerbated during male takeovers [21], [33]. However, herding behaviour in this context may reflect a genuine conflict of interest among the aggressor male and the to-be-abducted victim female (sexual conflict hypothesis) or may be a by-product of male-male aggression (male competition hypothesis) which is redirected towards the female. In fact, both processes may well be operating at the same time (see [20]).

The main findings in support of the sexual coercion hypothesis obtained in the present study are that only males who acquired females during the takeover event increased their herding rates; that these males’ aggressive herding targeted the abducted females only [21], [33]; and that male takeovers were detrimental only to the abducted females’ immediate reproductive prospects. That is just what one would expect if herding during male takeovers were used to condition and coerce the female into following and becoming attached to the aggressor male [21]. However, male takeovers typically involve a sequence of male-to-male aggression followed by male-to-female intense herding [21]. Therefore, herding in this context appears to be initially driven by male-male competition which eventually, but collaterally, might turn into redirected aggression towards the contested female. In any case, as already noted, the selectivity of the target of the male’s aggressive herding and the negative effects on the abducted female’ reproduction suggest that this functions as sexual coercion.

The few studies that have been conducted so far to test the sexual coercion hypothesis in primates have mainly addressed the issue of whether male aggression actually suppresses female mate choice [17], [18]. In this regard, the scenario created by male takeovers and aggressive herding of females in the hamadryas baboon is interesting for several reasons. First, male challengers tend to be physically more powerful than the contested females’ current unit male. Therefore, successful male takeovers might give females an opportunity to access a new mating partner with good genes, who is probably better able than its predecessor to provide fitness-enhancing services, especially protection against conspecific aggression and perhaps privileged access to food resources controlled by these males and related to the latter’s superior fighting skills. Second, although the abducted females’ mate choice is suppressed and their reproductive performance experiences an immediate setback, we do not know the long-term effects of male takeovers on the females’ lifetime reproductive success. Specifically, it would be important to determine if the short-term negative fitness consequences of male takeovers are cancelled out in the long run because the new males make a better contribution to the females’ long-term reproductive output.

In summary, this study shows that male takeovers in hamadryas baboons are reproductively costly to the abducted females, a finding that has also been recently reported in a study of wild hamadryas baboons [22]. Thus, taken together, these two studies lend support to the hypothesis that male takeovers and female transfers in this species represent a male reproductive strategy based on sexual coercion [9], [13], [16]. Our data also suggest that the costs experienced by abducted females are most probably caused by the male’s aggressive herding. However, although male herding behaviour in hamadryas baboons is known to limit the movement of females and the expression of their mate preferences, to our knowledge no study has addressed the possibility that the immediate reproductive cost incurred by abducted females as a consequence of a male takeover are cancelled out in the long run, as new males are better prepared than their deposed rivals to provide fitness-enhancing services to the abducted females. Also, although the expression of female choice of mates or even other social partners is dramatically reduced or suppressed altogether in this species by the male’s herding behaviour, it remains to be established what factors are assessed by males before initiating a takeover. Specifically, we do not know if they mainly take into account a candidate female’s current OMU leader’s fighting power (i.e. the rival male’s vulnerability) or if they also assess the target female’s mate preferences (i.e. the female’s resistance). Early experiments in this species revealed that females did have mate preferences and that these preferences influenced the males’ decisions to attempt a takeover [53].
